# Weight-bearing and mobilisation timing after hip fracture surgery in older adults: an international survey of clinicians' perspectives

**DOI:** 10.1007/s41999-025-01205-z

**Published:** 2025-04-18

**Authors:** Ruqayyah Y. Turabi, Matthew D. L. O’Connell, David Wyatt, Chris Bretherton, Simon Cannon, Celia L. Gregson, Iain Moppett, Lynn McNicoll, Katie Jane Sheehan

**Affiliations:** 1 Department of Population Health Sciences, School of Life Course and Population Sciences, King’s College London, London, UK; 2 Department of Physical Therapy, College of Nursing and Health Sciences, Jazan University, Jazan, Saudi Arabia; 3 Bone and Joint Health, Blizard Institute, Queen Mary University of London, London, UK; 4 Barts Health NHS Trust, London, UK; 5 Musculoskeletal Research Unit, Bristol Medical School, University of Bristol, Bristol, UK; 6 Anaesthesia and Critical Care, Injury, Recovery and Inflammation Sciences, Queen’s Medical Centre, The University of Nottingham, Nottingham, UK; 7 Division of Geriatrics, Warren Alpert Medical School of Brown University, Providence, Rhode Island, USA

**Keywords:** Neck of femur fracture, Rehabilitation, Orthogeriatric, Mobilization timing, Weight-bearing, International

## Abstract

**Purpose:**

This exploratory study aimed to describe international variations in mobilisation timing and weight-bearing after hip fracture surgery, focusing on differences between high-income countries (HICs) and low- and middle-income countries (LMICs) and identify the possible reasons for these variations.

**Methods:**

This international cross-sectional study was administered through an online, English-language, self-reported questionnaire. Healthcare professionals from various multidisciplinary teams were invited to participate via professional organisations, including the Fragility Fracture Network, social media, and through snowballing from key individual stakeholders.

**Results:**

A total of 389 healthcare professionals from 71 countries participated in the survey. Among them, 72.5% prescribed mobilisation the day after surgery, higher in HICs (79.1%) than LMICs (56.3%). Of those who prescribed early mobilisation, 38.3% achieved it 76–100% of the time, more frequently in HICs (42.9%) than LMICs (21.9%). Additionally, 73.5% prescribed unrestricted weight-bearing, also more common in HICs (86.3%) than LMICs (41%). For those who prescribed unrestricted weight-bearing, 50.4% achieved it 76–100% of the time, with HICs at 54.0% and LMICs at 31.9%. Multiple patient-related, process-related, and structure-related barriers were reported, with structure-related barriers more common in LMICs than HICs, underscoring the global complexities in implementing these practices.

**Conclusion:**

This study offers insights into global variations in the timing of mobilisation and weight-bearing after hip fracture surgery in older adults. It reveals disparities in postoperative services and resources between HICs and LMICs. Additionally, it establishes a foundation for future research and underscores the importance of international collaboration and knowledge sharing in enhancing postoperative care services.

**Supplementary Information:**

The online version contains supplementary material available at 10.1007/s41999-025-01205-z.

## Key summary points

**Aim:**

To explore global variations in mobilisation timing and weight-bearing following hip fracture surgery, as well as differences between high-income countries (HICs) and low- and middle-income countries (LMICs), and to identify potential barriers to these practices

**Findings:**

Although there is a tendency towards early mobilisation and unrestricted weight-bearing, variations exist both within HICs and LMICs. Early mobilisation and unrestricted weight-bearing were more commonly prescribed and achieved in HICs than in LMICs. Structure-related barriers were reported more frequently in LMICs, underscoring the global complexities in implementing these practices.

**Message:**

International collaboration is crucial to address disparities in postoperative care and improve outcomes for older adults after hip fracture surgery.

**Supplementary Information:**

The online version contains supplementary material available at 10.1007/s41999-025-01205-z.

## Introduction

Hip fractures in older adults are associated with complications such as persistent pain, poor mobility, reduced quality of life, and increased mortality [1, 2]. Their health and economic burden is increasing, with 9.58 million new cases globally reported in 2019 among individuals aged 55 and older [3–5]. Given the global population distribution in 2022, where about 15.7% live in high-income countries (HICs) and about 84.3% live in low- and middle-income countries (LMICs) [6], this study aims to explore inherent differences in practices globally and between these two economic regions.

Clinical guidelines, predominately formulated by healthcare authorities and professional bodies in HICs, advocate for early mobilisation (defined as the ability of patients to sit or stand out of bed by the day after surgery [7]) and immediate unrestricted weight-bearing to enhance recovery after hip fracture surgery [8]. These practices have been associated with improved functional outcomes, shortened hospitalisation, and reduced postoperative complications, such as pressure sores, deep vein thrombosis, pneumonia, delirium, and mortality [9–13], and are often monitored through audits [14, 15]. Successfully implementing these practices requires a planned approach to rehabilitation that leverages the multidisciplinary team.

Despite these recommendations, variation in the prescription of early mobilisation [7, 16, 17] and unrestricted weight-bearing [18, 19] has been reported. Previous reviews of the available evidence identified barriers to both early mobilisation and weight-bearing [20–22], yet little is known about how these barriers differ across diverse healthcare settings globally. Barriers at the care processes and structure level may disproportionately affect patients from LMICs compared to HICs. These disparities may stem from variations in healthcare infrastructure, resource allocation, and access to advanced medical technologies and skilled professionals, which are often limited in LMICs than in HICs [23, 24]. This highlights the need to examine global healthcare practices through the lens of economic and structural differences. Therefore, this exploratory study aimed to describe the extent of global variation in mobilisation timing and weight-bearing after hip fracture surgery, the achievement of early mobilisation and unrestricted weight-bearing, and to explore the possible underlying barriers to these practices, with a specific focus on HICs and LMICs.

## Methods

This study was reported according to Strengthening the reporting of observational studies in epidemiology (STROBE) [25]. It received institutional ethical approval from King’s College London (MRSP-22/23–36,307). It was designed to be completely anonymous, with no identifying information collected from respondents.

### Study design

A cross-sectional study delivered via an online English-language self-administered questionnaire with four sections and 18 questions. The questionnaire structure was informed by a previous global survey led by IM and LM [26], and the scope and questions were developed by RT, KS and DW based on a published scoping review [22].

### Data collection

Respondents were informed of their rights and provided written consent before completing the 5-min questionnaire, which used closed questions such as multiple-choice and multiple-answer questions to minimise the burden and increase the response rate. Multiple-choice questions allowed respondents to choose one answer from a list of options, while multiple-answer questions allowed for the selection of multiple responses. The questionnaire was piloted with two individuals before being refined. The refinement involved clarifying terms, rearranging choices and questions, and combining similar choices. The questionnaire collected data on several variables: respondent countries, timing of mobilisation, early mobilisation achievement, mobilisation data collection, weight-bearing prescriptions, unrestricted weight-bearing achievements, weight-bearing data collection method, barriers to prescribing and achieving early mobilisation, barriers to prescribing and achieving unrestricted weight-bearing, and details of respondents' institutions and specialities. The questionnaire is available in (Supplementary file S1).

### Sampling and study administration

Healthcare professionals (e.g. geriatricians, nurses, orthopaedic surgeons, physical therapists, or physicians) from around the globe who manage older adults undergoing hip fracture surgery were invited to participate. Eligibility required respondents to be involved in care for older adults who had hip fracture surgery. A convenience sampling approach was employed to collect as many responses as possible both within countries and globally.

An online Qualtrics questionnaire [27] was circulated on 28th March 2023 via email and social media to members of the Fragility Fracture Network (FFN). The FFN is a global organisation whose mission is to optimise globally the multidisciplinary management of patients with fragility fractures, including secondary prevention (https://fragilityfracturenetwork.org/). It was also promoted on social media, including X and LinkedIn. Additionally, snowballing from key stakeholders and professional bodies, including the World Confederation for Physical Therapy’s Database of Volunteers and Experts (DOVE) mailing list, which comprises international volunteers who help in developing the profession internationally, were approached to facilitate the circulation of the questionnaire through a wider network. FFN promoted the study with follow-up emails and at their global Congress between 4 and 6th October 2023, after which the study was closed.

### Descriptive statistics

Data was exported from Qualtrics to Stata (18.0 for Mac) [28] and Microsoft Excel [29] for quantitative analysis and data visualisation, respectively. Global maps were created by MapChart [30]. Data was cleaned, and responses that included only consent and country information were excluded from the analysis.

Responses were summarised as proportions overall and grouped by the World Bank Country Classification as HICs or LMICs [31]. These proportions were collected for the timing of mobilisation and weight-bearing prescriptions, achievement of early mobilisation, and achievement of unrestricted weight-bearing. Proportions for perceived barriers to prescribing and achieving these practices were also collected and further categorised into patient-, process- and structure-related barriers.

## Results

### Respondent characteristics

**Fig. 1:**
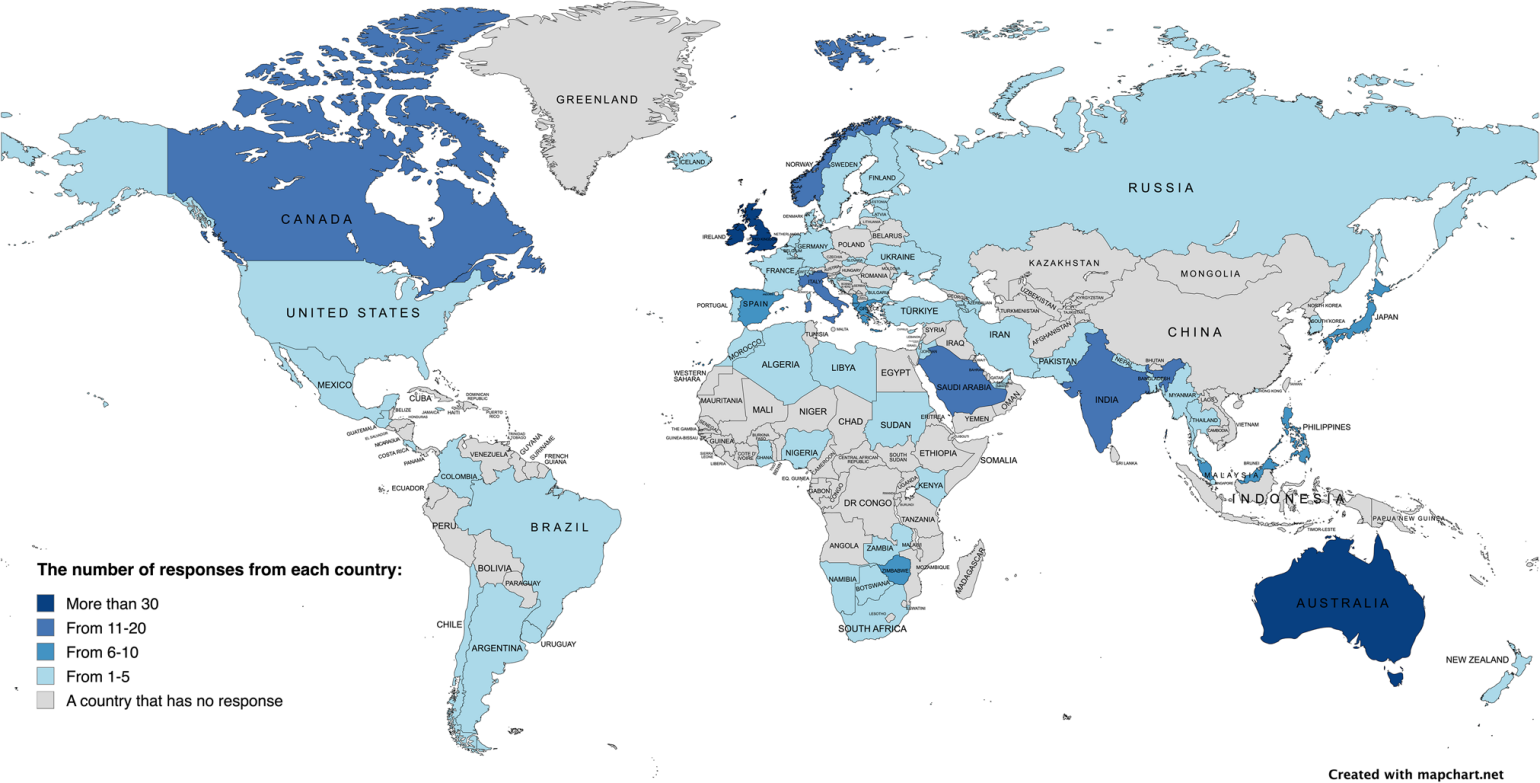
Responses from 389 respondents across 71 countries

Overall, 529 people from 81 countries accessed the questionnaire. Of them, 50 consented and did not answer any subsequent questions, and 90 consented and only answered their country of residence. These were excluded, leaving 389 respondents from 71 countries for analysis (36.4% of the world's countries) (Fig. 1). Of the respondents, 71.2% (n = 277) were from HICs and 28.8% (n = 112) were from LMICs. Overall, 274 (70.4%) responded to speciality and institution of work. Most respondents were orthopaedic surgeons (36.1%) or physical therapists (30.3%), followed by ortho-geriatricians (12.0%) and nurses (9.1%). Most respondents worked in acute teaching hospitals (60.2%), followed by acute non-teaching hospitals (13.9%), orthopaedic hospitals (8.0%), and higher education settings (5.8%). Table 1 summarises the respondents’ characteristics.

**Table 1:** Geographic regions, specialities, and institution types of study respondents

	Total, n (%)	High-income countries (HICs)^†^, n (%)	Low- and middle-income countries (MICs)^‡^, n (%)
Geographic regions* (n = 389)			
Northern Europe	127 (32.7)	127 (45.9)	–
Southern Europe	44 (11.3)	38 (13.7)	6 (5.4)
Oceania	39 (10)	39 (14.1)	–
Western Asia	32 (8.2)	24 (8.7)	8 (7.1)
Southern Asia	29 (7.5)	–	29 (25.9)
Latin America	25 (6.4)	7 (2.5)	18 (16.1)
Sub-Saharan Africa	20 (5.1)	–	20 (17.9)
South-eastern Asia	19 (4.9)	2 (0.7)	17 (15.2)
Northern America	15 (3.9)	15 (5.4)	–
Eastern Asia	12 (3.1)	12 (4.3)	–
Western Europe	12 (3.1)	12 (4.3)	–
Eastern Europe	10 (2.6)	1 (0.4)	9 (8.0)
Northern Africa	5 (1.3)	–	5 (4.5)
Total	389	277 (71.2)	112 (28.8)
Speciality (n = 274)^§^			
Orthopaedic surgeon	99 (36.1)	60 (30.6)	39 (50.0)
Physical therapist	83 (30.3)	58 (29.6)	25 (32.1)
Orthogeriatricians	33 (12.0)	31 (15.8)	2 (2.6)
Nurse	25 (9.1)	22 (11.2)	3 (3.9)
Specialist trainee geriatrician	8 (2.9)	6 (3.1)	2 (2.6)
Physician	6 (2.2)	3 (1.5)	3 (3.9)
Staff or associate specialist	3 (1.1)	2 (1.0)	1 (1.3)
Occupational therapist	2 (0.7)	2 (1.0)	–
Others^a^	15 (5.5)	12 (6.1)	3 (3.9)
Total	274	196	78
Institution type (n = 274)^§^			
Acute teaching hospital	165 (60.2)	135 (68.9)	30 (38.5)
Acute non-teaching hospital	38 (13.9)	27 (13.8)	11 (14.1)
Orthopaedic hospital	22 (8.0)	13 (6.6)	9 (11.5)
University/college	16 (5.8)	7 (3.6)	9 (11.5)
Outpatient rehabilitation centre	11 (4.0)	4 (2.0)	7 (9.0)
Government agency	7 (2.6)	3 (1.5)	4 (5.1)
Inpatient rehabilitation centre	5 (1.8)	4 (2.0)	1 (1.3)
Outpatient department	2 (0.7)	1 (0.5)	1 (1.2)
Others^b^	8 (2.9)	2 (1.02)	6 (7.7)
Total	274	196	78

*Geographic regions according to the United Nations [32]

^†^High-income countries (HICs): Australia, Belgium, Canada, Chile, Croatia, Cyprus, Denmark, Estonia, Finland, France, Germany, Greece, Hong Kong (S.A.R.), Iceland, Ireland, Israel, Italy, Jamaica, Japan, Latvia, Netherlands, New Zealand, Norway, Portugal, Saudi Arabia, Singapore, Slovakia, South Korea, Spain, Sweden, Switzerland, the United Arab Emirates, the United Kingdom of Great Britain and Northern Ireland, the United States of America, Uruguay

^‡^Low- and middle-income countries (MICs): Albania, Algeria, Argentina, Armenia, Azerbaijan, Bangladesh, Botswana, Brazil, Bulgaria, Colombia, Costa Rica, Ghana, Guatemala, India, Iran, Jordan, Kenya, Libyan Arab Jamahiriya, Malaysia, Mauritius, Mexico, Morocco, Myanmar, Namibia, Nepal, Nigeria, Pakistan, Philippines, Russian Federation, South Africa, Sudan, Thailand, Turkey, Ukraine, Zambia, Zimbabwe

^§^Completed responses where 115 responses were missing

^a^ Included anaesthetist (n = 1), consultant geriatrician (n = 1), geriatrician (n = 4), nurse practitioner (n = 2), nurse specialist in rehabilitation (n = 2), orthopaedic frailty advance nurse practitioner (n = 1), and physiatrist (n = 4)

^b^ Included private hospitals (n = 4), home and community care (n = 2), military hospital (n = 1), and both private and public hospitals (n = 1)

### Timing of mobilisation and early mobilisation achievement

Among the 389 respondents to the mobilisation time data source question, 52.4% reported 'best guess', with 63.4% from LMICs and 48.0% from HICs. 44.5% reported systematic audits or database reviews, with 49.8% from HICs and 31.3% from LMICs. Additionally, 3.1% reported 'other' methods, with 5.4% from LMICs and 2.2% from HICs.

**Fig. 2:**
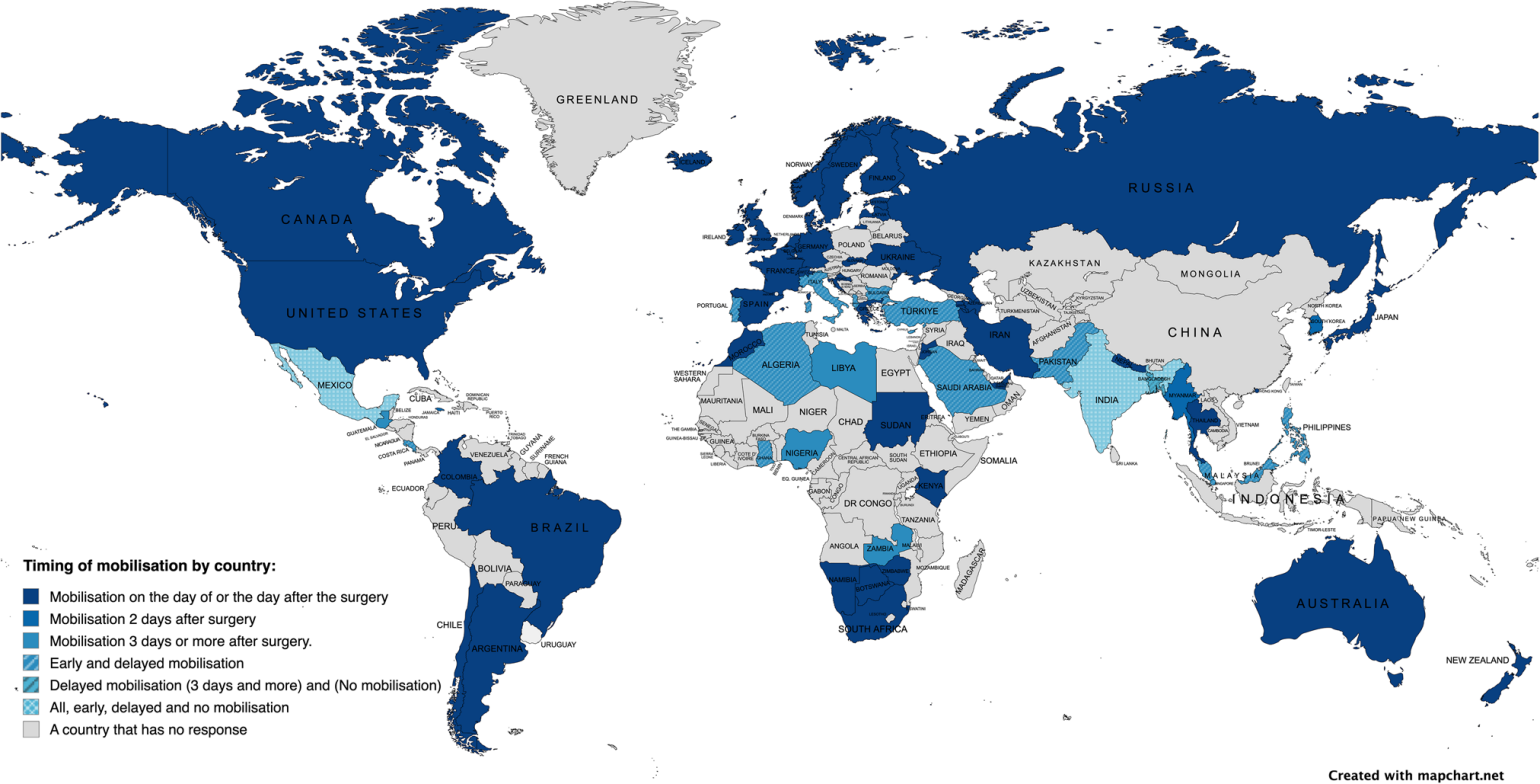
Response to ‘most frequent mobilisation timing prescription’ by country

**Fig. 3:**
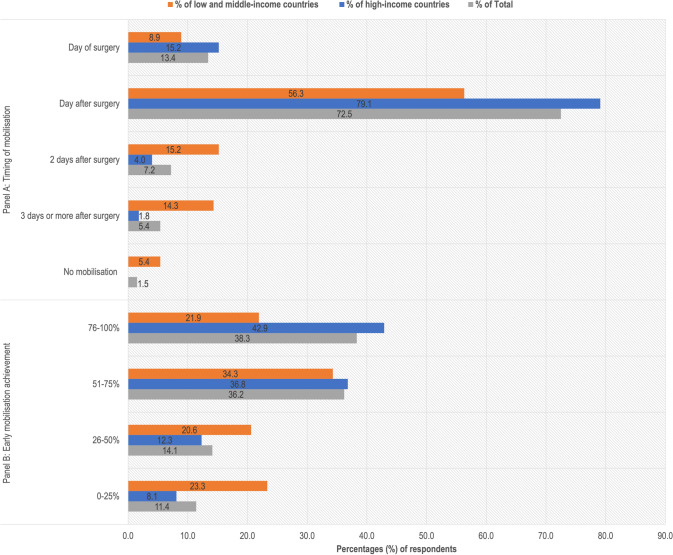
Mobilisation practices overall and by World Bank Country Classification as high-income, or low- and middle-income. Grey represents the percentage of total responses, blue represents the percentage of responses from high-income countries, and orange represents the percentage of responses from low- and middle-income countries. **A** Timing of mobilisation. **B** The extent to which mobilisation on the day of or day after surgery is achieved.

Overall, 389 responded to the question ‘Which mobilisation approach is the most common where you work?’. A global map (Fig. 2) presents the timing of mobilisation reported across different countries. 13.4% reported prescribing mobilisation on the day of surgery, 72.5% the day after surgery, 7.2% two days after surgery, 5.4% three or more days after surgery, and 1.5% indicated that no mobilisation was prescribed (Fig. 3A).

Out of the 389 respondents, 85.9% reported prescribing mobilisation on the day or the day after surgery. Among these respondents, 38.3% reported achieving early mobilisation between 76 and 100% of the time, 36.2% between 51 and 75% of the time, 14.1% between 26 and 50% of the time, and 11.4% between 0 and 25% of the time (Fig. 3B).

### Weight-bearing prescription and unrestricted weight-bearing achievement

Among the 389 respondents to the weight-bearing data source question, 58.9% reported 'best guess', 62.5% from LMICs and 57.4% from HICs. 38.6% reported systematic audits or database reviews, with 40.0% from HICs and 34.8% from LMICs. Additionally, 2.6% reported 'other' methods, with 2.7% from LMICs and 2.5% from HICs.

**Fig. 4:**
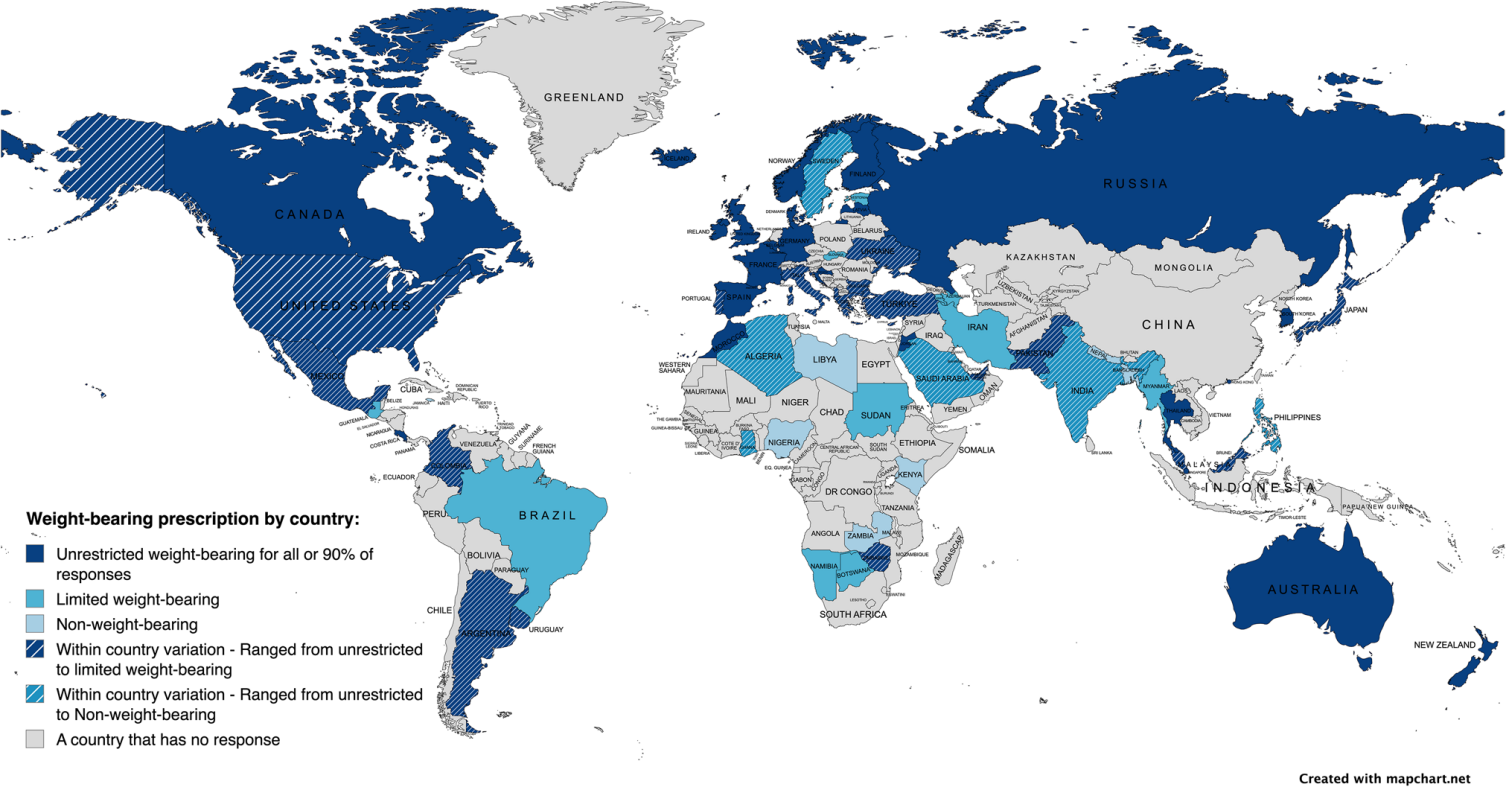
Response to ‘most frequent weight-bearing prescription’ by country

**Fig. 5:**
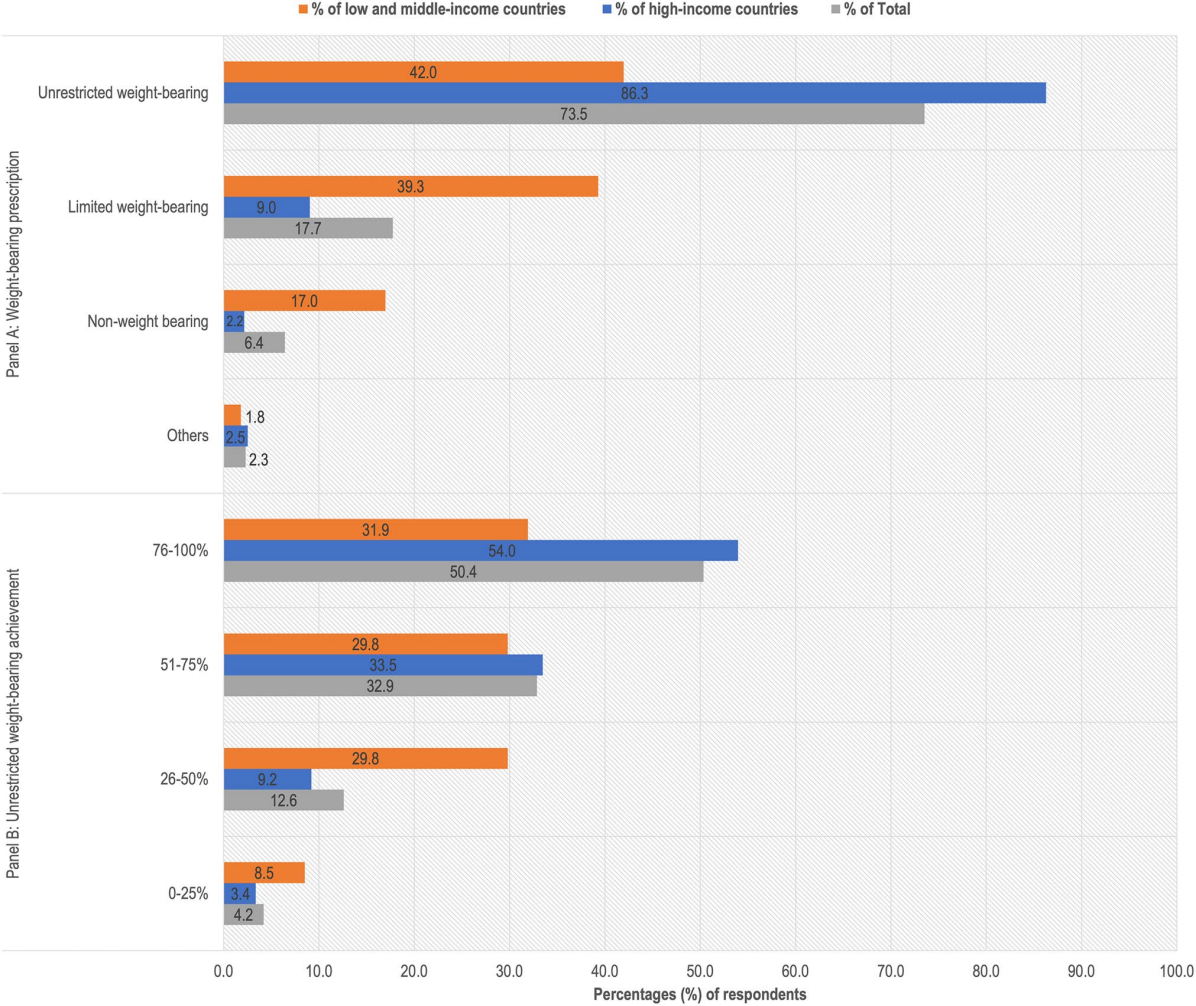
Weight-bearing practices overall and by World Bank Country Classification as high-income, or low- and middle-income. Grey represents the percentage of total responses, blue represents the percentage of responses from high-income countries, and orange represents the percentage of responses from low- and middle-income countries. **A** Prescription of weight-bearing. **B** The extent to which unrestricted weight-bearing on the day of or day after surgery is achieved.

Overall, 389 responded to the question ‘Which weight-bearing prescription is the most common after hip fracture surgery where you work?’. A global map (Fig. 4) presents the variation in weight-bearing prescription across different countries. 73.5% reported prescription of unrestricted weight-bearing, 17.7% limited weight-bearing, 6.4% non-weight-bearing, and 2.3% reported 'other'. 'Other' included: dependent on protocol, surgeon, fracture type, or surgery type (Fig. 5A).

Out of the 389 respondents, 73.5% reported prescribing unrestricted weight-bearing. Of these, 50.4% reported achieving unrestricted weight-bearing between 76 and 100% of the time, 32.9% between 51 and 75% of the time, 12.6% unrestricted weight-bearing between 26 and 50% of the time, and 4.2% between 0 and 25% of the time (Fig. 5B).

### Barriers

Barriers to early mobilisation and/or unrestricted weight-bearing were grouped into patient-related, process-related, and structure-related barriers (Tables S1, S2, S3 and S4).

### Barriers to prescribing early mobilisation

Overall, 351 respondents reported barriers to prescribing early mobilisation (Table S1). The most reported patient-related barriers were poor pre-fracture mobility levels (23.4%), frailty (21.4%), and dementia (19.1%). Process-related barriers included the type of anaesthesia (9.4%). Structure-related barriers included low staffing (19.9%) and insufficient resources or equipment (14.0%).

### Barriers to achieving early mobilisation

Overall, 333 respondents reported barriers to achieving early mobilisation (Table S2). The most reported patient-related barriers were uncontrolled pain (52.0%), patient refusal (51.1%), and postoperative delirium (44.7%). Process-related barriers were type of anaesthesia (9.9%), and long time to surgery (9.6%). Structure-related barriers included low staffing (29.1%), poor multidisciplinary engagement (17.1%) and insufficient resources or equipment (14.7%).

### Barriers to prescribing unrestricted weight-bearing

Overall, 310 respondents reported barriers to prescribing unrestricted weight-bearing (Table S3). The most reported patient-related barriers influencing weight-bearing prescriptions after surgery were the type of fracture (30.0%), followed by other intraoperative complications (19.7%), and frailty (15.8%). Process-related barriers included the type of surgery (35.2%), surgical approach (e.g., anterior, anterolateral, posterior) (13.2%), and the type of anaesthesia (6.5%). Structure-related barriers to prescribing unrestricted weight-bearing included low staffing (12.6%), and insufficient resources or equipment (8.4%).

### Barriers to achieving unrestricted weight-bearing

Overall, 284 respondents reported barriers to achieving unrestricted weight-bearing (Table S4). The highest reported patient-related barriers were uncontrolled pain (43.7%), patient refusal (43.0%), and postoperative delirium (36.3%). Process-related barriers included type of surgery (22.2%), surgical approach (7.7%), the type of anaesthesia (6.7%), and the long time to surgery (5.6%). Structure-related barriers included low staffing (19.4%), insufficient resources or equipment (13.7%), and low multidisciplinary engagement (11.3%).

## Discussion

This study is the first to explore global variations in the timing of mobilisation and weight-bearing prescriptions and the extent of their achievement after hip fracture surgery in older adults. It shows variations in these practices across 71 participating countries. While there is a tendency towards early mobilisation and unrestricted weight-bearing, with a majority prescribing mobilisation the day after surgery (73%), notable variations are evident within and between HICs and LMICs. In HICs, a predominant preference for early mobilisation on the day after surgery (79%) coexists with a minority prescribing mobilisation on the day of surgery (15%), illustrating diverse practices within similar economic contexts. Greater variation was observed for LMICs, although there was a tendency towards early mobilisation (56%). This variation in practices extended to weight-bearing prescriptions, where HICs predominantly reported unrestricted weight-bearing (86%), while LMICs reported a range of approaches, including limited weight-bearing (39%) and non-weight-bearing (17%). Further, the study identified perceived barriers to prescribing and achieving these practices, which predominately focused on patient-related factors with process- and structure-related factors reported less frequently. These factors also varied by World Bank Country Classification.

The results show reliance on ‘best guess’ internationally, for both mobilisation timing and weight-bearing prescription. Interestingly, systematic audits were more reported for collecting mobilisation data than for weight-bearing prescriptions. This was prominent even in HICs, where some established healthcare audits exist, which could suggest a de-prioritisation of weight-bearing as an indicator of care. Indeed, the minimum common dataset for hip fracture audit specifies mobilisation time but not weight-bearing prescription [14].

Our findings align with national audits in high-income settings, where early mobilisation and unrestricted weight-bearing are widely accepted. The National Hip Fracture Database (NHFD) reported that 81% of older adults with hip fractures were mobilised by the day after surgery, while the Irish Hip Fracture Database (IHFD) reported that 87% were mobilised on the day of or the day after surgery, closely matching our survey’s 79% in HICs [33, 34]. The Australian and New Zealand Hip Fracture Registry (ANZHFR) reported that 96% of patients in Australia and New Zealand were permitted unrestricted weight-bearing, closely aligning with our survey’s 86% in HICs [35]. Further, a UK multicentre audit found that 95% of patients were permitted unrestricted weight-bearing, reinforcing the consistency of this approach in HICs [15]. However, despite high prescription rates, only 39% in New Zealand and 48% in Australia achieved mobilisation on the first postoperative day, suggesting challenges in implementation [35]. These findings suggest that while early mobilisation and unrestricted weight-bearing are well-integrated into clinical protocols, achieving early mobilisation is yet a challenge. National registries play a key role in monitoring and improving outcomes, but inconsistencies in weight-bearing documentation may limit their impact, highlighting the need for expanded reporting to enhance standardisation and rehabilitation strategies.

The prescription of early mobilisation following hip fracture surgery was found to be affected by several key factors. The most reported barriers identified were non-modifiable patient-related such as poor pre-fracture mobility levels, frailty, and dementia. Notably, these barriers were more frequently reported in LMICs than in HICs, possibly reflecting differences in experience with rehabilitation of older adults with frailty [24, 36]. Beyond these barriers, respondents from LMICs more often reported structure-related barriers, including staffing shortages and a lack of necessary resources and equipment. This aligns with the findings from Armstrong and colleagues who explored barriers to, and enablers of, evidence-informed hip fracture care in LMICs [23]. This might be exacerbated by the "brain drain" phenomenon through the migration of healthcare professionals from LMICs to HICs, with better incentives, thus intensifying the resource gap and widening global healthcare disparities [24, 36]. This dichotomy between HICs and LMICs underscores the complexity of prescribing early mobilisation and necessitates a multifaceted approach to improve postoperative care for older adults with hip fractures [23].

In contrast to early mobilisation, this study found that process-related factors, primarily the type of surgery, were the most frequently reported barriers to prescribing unrestricted weight-bearing. This observation is consistent with existing literature [22], which indicates that the surgical approach, including the decision between fixation and arthroplasty, plays a considerable role in determining the perceived feasibility of prescribing unrestricted weight-bearing. However, this contrasts with international guidelines that explicitly recommend unrestricted weight-bearing, irrespective of surgery type, since modern implants are designed to be load-sharing devices, allowing unrestricted weight-bearing [16, 37]. Additionally, patient-related factors, such as the type of fracture and the presence of intraoperative periprosthetic complications, were also identified as crucial determinants of weight-bearing prescriptions. Consequently, this study highlights the need for a nuanced understanding and application of weight-bearing protocols after surgery, considering specific surgical approaches and patient-related factors, and underscores the ongoing debate in the literature. These findings call for additional research to reconcile varying viewpoints and establish a definitive, evidence-based approach that aligns with clinical guidelines to optimise patients’ outcomes after surgery.

The most reported barriers to achieving both early mobilisation and unrestricted weight-bearing were modifiable patient-related factors, namely uncontrolled pain and patient refusal, possibly reflecting the current knowledge in the area [20, 38]. This study shows that uncontrolled pain was the most frequently reported barrier in LMICs, possibly reflecting disparities in access to effective pain management resources such as analgesics and healthcare infrastructure [39]. Meanwhile, patient refusal was the most frequently reported barrier in HICs, potentially indicative of different patient expectations, possibly influenced by greater access to healthcare information and a cultural emphasis on autonomy. Pain, being a subjective and complex experience, can profoundly inhibit a patient’s willingness and ability to participate in early mobilisation, despite the known benefits for recovery. Pain not only hinders willingness to participate in early mobilisation and weight-bearing activities but also leads to fear avoidance behaviour [40], which may contribute to patient refusal. This cycle of pain and reluctance to engage in mobility activities underscores the need for a holistic approach in postoperative care that addresses both the physical and psychological aspects of recovery to prevent a self-perpetuating cycle of pain and reduced mobility.

Healthcare professionals in HICs also more frequently reported postoperative delirium, symptomatic hypotension, and anaemia as barriers to achieving early mobilisation when compared to those in LMICs. These findings align with previous national sprint audits of physiotherapy after hip fracture surgery [16, 41]. While these barriers are inherently patient-related, their frequency and impact may also be influenced by healthcare practices. Higher reporting rates in HICs may reflect variations in reporting practices or healthcare resources, including more comprehensive monitoring, documentation protocols, and differences in clinical management strategies such as transfusion thresholds or access to blood products [42]. These findings highlight the need for further investigation to determine whether the observed disparities reflect true differences in incidence or variations in detection and management.

This study highlights the variability in mobilisation and weight-bearing practices after hip fracture surgery and suggests that further research is warranted, particularly in underrepresented LMICs. Addressing these variations may require inclusive international collaborations to understand underlying barriers and promote evidence-based practices tailored to varied healthcare settings. These insights could facilitate targeted discussions on resource allocation, training opportunities, and policy adjustment to reduce disparities and improve postoperative care. As part of these efforts, the FFN should continue to promote best practices for fragility fracture care, including early mobilisation and unrestricted weight-bearing, through position papers, education resources, offering forums for open discussion, and sharing of best practices through National FFNs. Further engagement with global health organisations and decision-makers could enhance the adoption and adaptation of mobilisation strategies across diverse healthcare systems. Ensuring adequate rehabilitation resources and integrating mobilisation into standard postoperative care policies could help bridge existing disparities and promote more equitable recovery outcomes worldwide.

This study has some limitations. Sampling bias may have resulted from the convenience sampling approach and the uneven response rate, with higher representation from the United Kingdom, Australia, and Ireland. While expansive, dissemination through FFN and DOVE may have underestimated global variation by reaching healthcare professionals in evidence-based networks. Categorising countries to HICs and LMICs, while practical, may have masked the variations within healthcare systems. Self-reported data may introduce interpretation bias, compounded by potential misinterpretation of survey items due to language and cultural differences.

Finally, as an exploratory study, the findings are not intended to establish generalisability but rather to be seen as hypotheses-generating and provide a foundation for future research aimed at addressing global disparities and improving mobility recovery strategies globally.

## Conclusion

This study offers insights into global variations in the timing of mobilisation and weight-bearing prescriptions, as well as the achievement of early mobilisation and unrestricted weight-bearing following hip fracture surgery in older adults. While international guidelines advocate for early mobilisation and unrestricted weight-bearing as crucial steps to recovery of mobility after surgery, this study highlights international variations in these practices and underscores the distinct disparities in services, practices, and resources between HICs and LMICs. The study also sheds light on various patient-, process- and structure-related barriers that are perceived to influence these practices. Specifically, respondents viewed the mobilisation timing to be predominantly influenced by patient barriers, while weight-bearing was seen to be influenced by process barriers. Moreover, structure-related barriers were more reported in LMICs than in HICs. This study underscores the need for consistent recording practices and context-specific strategies in postoperative care, acknowledging the variability in practices and resources across different regions. These findings lay the groundwork for future studies and highlight the importance of international collaboration and knowledge exchange in improving postoperative outcomes for older adults with hip fractures globally.

## Supplementary Information

Below is the link to the electronic supplementary material.

Supplementary file1 (DOCX 29 KB)

Supplementary file2 (DOCX 35 KB)
