# Supplementary material for: Weight-bearing and mobilisation timing after hip fracture surgery in older adults: an international survey of clinicians' perspectives
Source: Eur Geriatr Med. 2025 Apr 18;16(3):909–20. doi: 10.1007/s41999-025-01205-z (PMC12174268; doi:10.1007/s41999-025-01205-z)
Supplement: Supplementary file 1 — Supplementary file1 (DOCX 29 KB) [file 41999_2025_1205_MOESM1_ESM.docx]

**Weight-bearing and Mobilisation timing after hip fracture surgery in older adults: An international survey of clinicians' perspectives.**

Ruqayyah Y Turabi^1,2^*, Matthew DL O'Connell^1^, David Wyatt^1^, Chris Bretherton^3^, Simon Cannon^4^, Celia L Gregson^5^, Iain Moppett^6^, Lynn McNicoll^7^, Katie Jane Sheehan^3^

^1^ Department of Population Health Sciences, School of Life Course and Population Sciences, King's College London, London, United Kingdom

^2^ Department of Physical Therapy, College of Nursing and Health Sciences, Jazan University, Jazan, Saudi Arabia

^3^ Bone and Joint Health, Blizard Institute, Queen Mary University of London, London, United Kingdom

^4^ Barts Health NHS Trust, London, United Kingdom

^5^ Musculoskeletal Research Unit, Bristol Medical School, University of Bristol, Bristol, United Kingdom

^6^ Anaesthesia and Critical Care, Injury, Recovery & Inflammation Sciences, The University of Nottingham, Queen’s Medical Centre, Nottingham, United Kingdom

^7^ Division of Geriatrics, Warren Alpert Medical School of Brown University, Providence, Rhode Island, USA

*Corresponding author:

Ruqayyah Y Turabi

[Ruqayyah.turabi@kcl.ac.uk](mailto:Ruqayyah.turabi@kcl.ac.uk)

ORCID ID: <https://orcid.org/0000-0001-6439-6276>

# Supplementary File (S1)

**Section 1:**

1. In which country do you work?

**What is happening where you work?**

**For the purposes of this survey, mobilisation is defined by the ability to sit or stand out of bed by the day after the surgery (standing or by hoist)**

1. Which mobilisation approach is the most common where you work?

- Mobilisation on the day of the surgery
- Mobilisation on the day after the surgery.
- Mobilisation 2 days after surgery.
- Mobilisation 3 days or more after surgery.
- No mobilisation offered.

1. How often is “mobilisation on the day of surgery or the day after surgery” **achieved** by patients where you work?

- 0-25%
- 26-50%
- 51%-75%
- 76%-100%

1. Is this based on systematic audit/data collection or a ‘best guess’?

- Systematic audit/data collection
- Best guess
- Other (please specify)

1. Which weight-bearing prescription is the most common after hip fracture surgery where you work?

- Weight bearing as tolerated/full weight bearing.
- Partial/toe-touch weight bearing.
- Non-weight bearing.
- Other (Please specify)

1. How often is “weight bearing as tolerated” **achieved** by patients where you work?

- 0-25%
- 26-50%
- 51%-75%
- 76%-100%

**Section 2:**

**What is preventing people from mobilising on the day of surgery or the day after where you work (as applicable)?**

1. Is this based on systematic audit/data collection or a ‘best guess’?

- Systematic audit/data collection
- Best guess
- Other (please specify)

1. If “mobilisation on the day of surgery or the day after surgery” is **not prescribed** for all patients where you work, why not? (Please select **all that apply**)

- Staffing issues (availability of staff to support)
- Insufficient resources/equipment
- Type of anaesthesia
- ASA score
- Preoperative delirium
- Infection
- Pre-fracture mobility level
- Pre-fracture residence in a care home
- Frailty
- Dementia
- Malignancy/Cancer
- Acute kidney injury
- Chronic kidney disease
- Cardiovascular disease
- Short life expectancy
- Previous hip fracture
- Other (please specify)

1. If “mobilisation on the day of surgery or the day after surgery” is **not prescribed** for all patients where you work, why not? (Please select **top 5 reasons**)

- Staffing issues (availability of staff to support)
- Insufficient resources/equipment
- Type of anaesthesia
- ASA score
- Preoperative delirium
- Infection
- Pre-fracture mobility level
- Pre-fracture residence in a care home
- Frailty
- Dementia
- Malignancy/Cancer
- Acute kidney injury
- Chronic kidney disease
- Cardiovascular disease
- Short life expectancy
- Previous hip fracture
- Other (please specify)

1. If “mobilisation on the day of surgery or the day after surgery” is **not achieved** for all patients where you work, why not? (Please select **all that apply**)

- Staffing issues (availability of staff to support)
- Low multidisciplinary engagement
- Insufficient resources/equipment
- Time to surgery
- Type of anaesthesia
- Preoperative delirium
- ASA score
- Infection
- Patient refusal
- Postoperative delirium
- Pain control issues
- Electrolyte imbalance
- Anaemia
- Tachycardia
- Symptomatic hypotension
- Pre-fracture mobility level
- Pre-fracture residence in a care home
- Frailty
- Dementia
- Depression
- Malignancy/Cancer
- Acute kidney injury
- Chronic kidney disease
- Cardiovascular disease
- Short life expectancy
- Previous hip fracture
- Other (please specify)

1. If “mobilisation on the day of surgery or the day after surgery” is **not achieved** for all patients where you work, why not? (Please select **top 5 reasons**)

- Staffing issues (availability of staff to support)
- Low multidisciplinary engagement
- Insufficient resources/equipment
- Time to surgery
- Type of anaesthesia
- Preoperative delirium
- ASA score
- Infection
- Patient refusal
- Postoperative delirium
- Pain control issues
- Electrolyte imbalance
- Anaemia
- Tachycardia
- Symptomatic hypotension
- Pre-fracture mobility level
- Pre-fracture residence in a care home
- Frailty
- Dementia
- Depression
- Malignancy/Cancer
- Acute kidney injury
- Chronic kidney disease
- Cardiovascular disease
- Short life expectancy
- Previous hip fracture
- Other (please specify)

**Section 3:**

**What is preventing people from ‘weight bearing as tolerated’ where you work (as applicable)?**

1. If “weight bearing as tolerated” is **not prescribed** for all patients where you work. Why **not**? (Please select **all that apply**)

- Staffing issues (availability of staff to support)
- Insufficient resources/equipment
- Type of surgery (e.g., fixation, hemi-arthroplasty, arthroplasty)
- Surgical approach (e.g., anterior, anterolateral, posterior)
- Other intraoperative complications
- Type of anaesthesia
- Preoperative delirium
- ASA score
- Type of fracture
- Infection
- Pre-fracture mobility level
- Pre-fracture residence in a care home
- Frailty
- Increased body mass index
- Decreased body mass index/ malnutrition.
- Dementia
- Malignancy/Cancer
- Acute kidney injury
- Chronic kidney disease
- Cardiovascular disease
- Short life expectancy
- Previous hip fracture
- Other (please specify)

1. If “weight bearing as tolerated” is **not prescribed** for all patients where you work. Why **not**? (Please select **top 5 reasons**)

- Staffing issues (availability of staff to support)
- Insufficient resources/equipment
- Type of surgery (e.g., fixation, hemi-arthroplasty, arthroplasty)
- Surgical approach (e.g., anterior, anterolateral, posterior)
- Other intraoperative complications
- Type of anaesthesia
- Preoperative delirium
- ASA score
- Type of fracture
- Infection
- Pre-fracture mobility level
- Pre-fracture residence in a care home
- Frailty
- Increased body mass index
- Decreased body mass index/ malnutrition.
- Dementia
- Malignancy/Cancer
- Acute kidney injury
- Chronic kidney disease
- Cardiovascular disease
- Short life expectancy
- Previous hip fracture
- Other (please specify)

1. If weight bearing as tolerated is **not achieved** by all patients where you work. Why **not**? (Please select **all that apply**)

- Staffing issues (availability of staff to support)
- Low multidisciplinary engagement
- Insufficient resources/equipment
- Type of surgery (e.g., fixation, hemi-arthroplasty, arthroplasty)
- Surgical approach (e.g., anterior, anterolateral, posterior)
- Other intraoperative complications
- Time to surgery
- Type of anaesthesia
- Preoperative delirium
- ASA score
- Postoperative delirium
- Type of fracture
- Infection
- Patient refusal
- Pain control issues
- Symptomatic hypotension
- Pre-fracture mobility level
- Pre-fracture residence in a care home
- Frailty
- Increased body mass index
- Decreased body mass index/ malnutrition.
- Dementia
- Depression
- Malignancy/Cancer
- Acute kidney injury
- Chronic kidney disease
- Cardiovascular disease
- Short life expectancy
- Previous hip fracture
- Other (please specify)

1. If weight bearing as tolerated is **not achieved** by all patients where you work. Why **not**? (Please select **top 5 reasons**)

- Staffing issues (availability of staff to support)
- Low multidisciplinary engagement
- Insufficient resources/equipment
- Type of surgery (e.g., fixation, hemi-arthroplasty, arthroplasty)
- Surgical approach (e.g., anterior, anterolateral, posterior)
- Other intraoperative complications
- Time to surgery
- Type of anaesthesia
- Preoperative delirium
- ASA score
- Postoperative delirium
- Type of fracture
- Infection
- Patient refusal
- Pain control issues
- Symptomatic hypotension
- Pre-fracture mobility level
- Pre-fracture residence in a care home
- Frailty
- Increased body mass index
- Decreased body mass index/ malnutrition.
- Dementia
- Depression
- Malignancy/Cancer
- Acute kidney injury
- Chronic kidney disease
- Cardiovascular disease
- Short life expectancy
- Previous hip fracture
- Other (please specify)

1. Is there anything else you would like to tell us about your experiences related to weight bearing or mobilisation timing after hip fracture?

**Section 4:**

**About you**

1. Which of the following best describes where you work?

- Acute teaching hospital
- Acute non-teaching hospital
- Inpatient rehabilitation centre
- Orthopaedic hospital
- Outpatient department
- Outpatient rehabilitation centre
- Long-term care facility/nursing home/residential care
- University/college
- Government agency
- Others (Please specify)

1. What is your speciality?

- Orthopaedic surgeon
- Orthopaedic geriatrician
- Staff or associated specialist
- specialist trainee geriatrician
- Physician
- Nurse
- Physical therapist
- Occupational therapist
- Others (please specify)
