# Supplementary material for: Weight-bearing and mobilisation timing after hip fracture surgery in older adults: an international survey of clinicians' perspectives
Source: Eur Geriatr Med. 2025 Apr 18;16(3):909–20. doi: 10.1007/s41999-025-01205-z (PMC12174268; doi:10.1007/s41999-025-01205-z)
Supplement: Supplementary file 2 — Supplementary file2 (DOCX 35 KB) [file 41999_2025_1205_MOESM2_ESM.docx]

**Weight-bearing and Mobilisation timing after hip fracture surgery in older adults: An international survey of clinicians' perspectives.**

Ruqayyah Y Turabi^1,2^*, Matthew DL O'Connell^1^, David Wyatt^1^, Chris Bretherton^3^, Simon Cannon^4^, Celia L Gregson^5^, Iain Moppett^6^, Lynn McNicoll^7^, Katie Jane Sheehan^3^

^1^ Department of Population Health Sciences, School of Life Course and Population Sciences, King's College London, London, United Kingdom

^2^ Department of Physical Therapy, College of Nursing and Health Sciences, Jazan University, Jazan, Saudi Arabia

^3^ Bone and Joint Health, Blizard Institute, Queen Mary University of London, London, United Kingdom

^4^ Barts Health NHS Trust, London, United Kingdom

^5^ Musculoskeletal Research Unit, Bristol Medical School, University of Bristol, Bristol, United Kingdom

^6^ Anaesthesia and Critical Care, Injury, Recovery & Inflammation Sciences, The University of Nottingham, Queen’s Medical Centre, Nottingham, United Kingdom

^7^ Division of Geriatrics, Warren Alpert Medical School of Brown University, Providence, Rhode Island, USA

*Corresponding author:

Ruqayyah Y Turabi

[Ruqayyah.turabi@kcl.ac.uk](mailto:Ruqayyah.turabi@kcl.ac.uk)

ORCID ID: <https://orcid.org/0000-0001-6439-6276>

# **Supplementary File (S2)**

Table 1. Barriers to prescribing early mobilisation overall and by World Bank Country Classification as high income, or low- and middle-income.

| Categories | Barriers | Total (N=351)  n (%) | High-income country, (N=251)  n (%) | Low- and middle-income country (N=100)  n (%) |
| --- | --- | --- | --- | --- |
| Patient | Pre-fracture mobility level | 82.0 (23.4) | 45.0 (17.9) | 37.0 (37.0) |
|  | Frailty | 75.0 (21.4) | 44.0 (17.5) | 31.0 (31.0) |
|  | Dementia | 67.0 (19.1) | 44.0 (17.5) | 23.0 (23.0) |
|  | Preoperative delirium | 47.0 (13.4) | 30.0 (12.0) | 17.0 (17.0) |
|  | Cardiovascular disease | 37.0 (10.5) | 19.0 (7.6) | 18.0 (18.0) |
|  | Infection | 40.0 (11.4) | 25.0 (10.0) | 15.0 (15.0) |
|  | Short life expectancy | 21.0 (6.0) | 14.0 (5.6) | 7.0 (7.0) |
|  | American Society of Anaesthesiology (ASA) score | 18.0 (5.1) | 8.0 (3.2) | 10.0 (10.0) |
|  | Malignancy/Cancer | 18.0 (5.1) | 11.0 (4.4) | 7.0 (7.0) |
|  | Acute kidney injury | 16.0 (4.6) | 9.0 (3.6) | 7.0 (7.0) |
|  | Chronic kidney disease | 15.0 (4.3) | 7.0 (2.8) | 8.0 (8.0) |
|  | Pre-fracture residence in a care home | 15.0 (4.3) | 8.0 (3.2) | 7.0 (7.0) |
|  | Previous hip fracture | 13.0 (3.7) | 7.0 (2.8) | 6.0 (6.0) |
|  | Other* | 28 (8.0) | 25 (10.0) | 3 (3.0) |
| Process | Type of anaesthesia | 33.0 (9.4) | 17.0 (6.8) | 16.0 (16.0) |
|  | Other† | 14 (4.0) | 8 (3.2) | 6 (6.0) |
| Structure | Staffing issues (availability of staff to support) | 70.0 (19.9) | 39.0 (15.5) | 31.0 (31.0) |
|  | Insufficient resources/equipment | 49.0 (14.0) | 24.0 (9.6) | 25.0 (25.0) |

* Included type of fracture pain, high risk falls, osteoporosis, and unstable health status.

† Included type of surgery and surgeons' instructions, and late referrals to rehabilitation or waiting for x-rays.

Table 2. Barriers to achieving early mobilisation overall and by World Bank Country Classification as high income, or low- and middle-income.

| Categories | Barriers | Total (N=333)  n (%) | High-income country, (N=237)  n (%) | Low- and middle-income country (N=96)  n (%) |
| --- | --- | --- | --- | --- |
| Patient | Uncontrolled pain | 173 (52.0) | 119 (50.2) | 54 (56.3) |
|  | Patient refusal | 170 (51.1) | 127 (53.6) | 43 (44.8) |
|  | Postoperative delirium | 149 (44.7) | 120 (50.6) | 29 (30.2) |
|  | Symptomatic hypotension | 143 (42.9) | 115 (48.5) | 28 (29.2) |
|  | Pre-fracture mobility level | 103 (30.9) | 72 (30.4) | 31 (32.3) |
|  | Dementia | 97 (29.1) | 75 (31.6) | 22 (22.9) |
|  | Frailty | 85 (25.5) | 53 (22.4) | 32 (33.3) |
|  | Anaemia | 77 (23.1) | 62 (26.2) | 15 (15.6) |
|  | Preoperative delirium | 44 (13.2) | 30 (12.7) | 14 (14.6) |
|  | Tachycardia | 43 (12.9) | 26 (11.0) | 17 (17.7) |
|  | Cardiovascular disease | 41 (12.3) | 24 (11.0) | 17 (17.7) |
|  | Infection | 37 (11.1) | 24 (10.1) | 13 (13.5) |
|  | Electrolyte imbalance | 36 (10.8) | 22 (9.3) | 14 (14.6) |
|  | Depression | 28(8.4) | 16 (6.8) | 12 (12.5) |
|  | Short life expectancy | 25 (7.5) | 19 (8.0) | 6 (6.3) |
|  | Previous hip fracture | 16 (4.8) | 6 (2.5) | 10 (10.4) |
|  | Pre-fracture residence in a care home | 15 (4.5) | 9 (3.8) | 6 (6.3) |
|  | Malignancy/Cancer | 13 (3.9) | 6 (2.5) | 7 (7.3) |
|  | Acute kidney injury | 13 (3.9) | 7 (3.0) | 6 (6.3) |
|  | American Society of Anaesthesiology (ASA) score | 13 (3.9) | 8 (3.4) | 5 (5.2) |
|  | Chronic kidney disease | 11 (3.3) | 5 (2.1) | 6 (6.3) |
|  | Other* | 15 (4.5) | 14 (5.9) | 1 (1.0) |
| Process | Type of anaesthesia | 33 (9.9) | 18 (7.6) | 15 (15.6) |
|  | Time to surgery | 32 (9.6) | 18 (7.6) | 14 (14.6) |
|  | Other† | 4 (1.2) | 3 (1.3) | 1 (1.0) |
| Structure | Staffing issues (availability of staff to support) | 97 (29.1) | 67 (28.3) | 30 (31.3) |
|  | Low multidisciplinary engagement | 57 (17.1) | 24 (10.1) | 33 (34.4) |
|  | Insufficient resources/equipment | 49 (14.7) | 27 (11.4) | 22 (22.9) |
|  | Other‡ | 3 (0.9) | 3 (1.3) | 0 |

* Included type of fracture, pain, risk factors consideration (i.e., high risk falls, and fear of falling), and unstable general health and status (i.e., unstable vital signs, postoperative nausea, palliative care, impaired cognition, pre-fracture mobility level, and intubated patients in intensive care units and requiring high care in high dependency units).

†Included surgeons' instructions, late referrals to rehabilitation, conflicting operative notes and postoperative instructions, and waiting for x-rays

‡ Included availability of staff during weekend or holiday time and difficulties in identifying physiotherapist staff on weekends

Table 3. Barriers to prescribing unrestricted weight-bearing prescription overall and World Bank Country Classification as high income, or low- and middle-income.

| Categories | Barriers | Total (N=310)  n (%) | High-income country, (N=224)  n (%) | Low- and middle-income country (N=86)  n (%) |
| --- | --- | --- | --- | --- |
| Patient | Type of fracture | 93 (30.0) | 62 (27.7) | 31 (36.0) |
|  | Other intraoperative complications | 61 (19.7) | 42 (18.8) | 19 (22.1) |
|  | Frailty | 49 (15.8) | 27 (12.1) | 22 (25.6) |
|  | Pre-fracture mobility level | 42 (13.5) | 25 (11.2) | 17 (19.8) |
|  | Dementia | 40 (12.9) | 20 (8.9) | 20 (23.3) |
|  | Increased body mass index | 26 (8.4) | 15 (6.7) | 11 (12.8) |
|  | Infection | 22 (7.1) | 15 (6.7) | 7 (8.1) |
|  | Cardiovascular disease | 21 (6.8) | 11 (4.9) | 10 (11.6) |
|  | Decreased body mass index/ malnutrition | 19 (6.1) | 9 (4.0) | 10 (11.6) |
|  | Previous hip fracture | 17 (5.5) | 10 (4.5) | 7 (8.1) |
|  | Malignancy/Cancer | 13 (4.2) | 7 (3.1) | 6 (7.0) |
|  | Preoperative delirium | 13 (4.2) | 8 (3.6) | 5 (5.8) |
|  | Short life expectancy | 12 (3.9) | 5 (2.2) | 7 (8.1) |
|  | Pre-fracture residence in a care home | 12 (3.9) | 6 (2.7) | 6 (7.0) |
|  | American Society of Anaesthesiology (ASA) score | 9 (2.9) | 5 (2.2) | 4 (4.7) |
|  | Acute kidney injury | 8 (2.6) | 3 (1.3) | 5 (5.8) |
|  | Chronic kidney disease | 6 (1.9) | 2 (0.9) | 4 (4.7) |
|  | Other* | 12 (3.9) | 11 (4.9) | 1 (1.2) |
| Process | Type of surgery (e.g., fixation, hemi-arthroplasty, arthroplasty) | 109 (35.2) | 75 (33.5) | 34 (39.5) |
|  | Surgical approach (e.g., anterior, anterolateral, posterior) | 41 (13.2) | 25 (11.2) | 16 (18.6) |
|  | Type of anaesthesia | 20 (6.5) | 12 (5.4) | 8 (9.3) |
|  | Other† | 12 (3.9) | 12 (3.6) | 0 |
| Structure | Staffing issues (availability of staff to support) | 39 (12.6) | 15 (6.7) | 24 (27.9) |
|  | Insufficient resources/equipment | 26 (8.4) | 11 (4.9) | 15 (17.4) |

* Included type of fracture, Periprosthetic fracture, pain, high risk falls, osteoporosis, and fear of falling.

† Included surgeon preference, experience, protocols, conservative approaches by junior staff, fear of mechanical failure, unclear or undocumented weight-bearing orders, and surgeon availability for procedures like hemi or total arthroplasty

Table 4. Barriers to achieving unrestricted weight-bearing overall and by World Bank Country Classification as high income, or low- and middle-income.

| Categories | Barriers | Total (N=284)  n (%) | High-income country, (N=204)  n (%) | Low- and middle-income country (N=80)  n (%) |
| --- | --- | --- | --- | --- |
| Patient | Uncontrolled pain | 124 (43.7) | 93 (45.6) | 31 (38.8) |
|  | Patient refusal | 122 (43.0) | 92 (45.1) | 30 (37.5) |
|  | Postoperative delirium | 103 (36.3) | 78 (38.2) | 25 (31.3) |
|  | Dementia | 83 (29.2) | 66 (32.4) | 17 (21.3) |
|  | Symptomatic hypotension | 76 (26.8) | 60 (29.4) | 16 (20.0) |
|  | Frailty | 74 (26.1) | 48 (23.5) | 26 (32.5) |
|  | Other intraoperative complications | 62 (21.8) | 40 (19.6) | 22 (27.5) |
|  | Pre-fracture mobility level | 60 (21.1) | 44 (21.6) | 16 (20.0) |
|  | Type of fracture | 56 (19.7) | 36 (17.6) | 20 (25.0) |
|  | Preoperative delirium | 29 (10.2) | 20 (9.8) | 9 (11.3) |
|  | Depression | 27 (9.5) | 15 (7.4) | 12 (15.0) |
|  | Infection | 25 (8.8) | 13 (6.4) | 12 (15.0) |
|  | Increased body mass index | 23 (8.1) | 12 (5.9) | 11 (13.8) |
|  | Cardiovascular disease | 21 (7.4) | 13 (6.4) | 8 (10.0) |
|  | Decreased body mass index/ malnutrition | 16 (5.6) | 9 (4.4) | 7 (8.8) |
|  | Acute kidney injury | 15 (5.3) | 9 (4.4) | 6 (7.5) |
|  | Pre-fracture residence in a care home | 14 (4.9) | 10 (4.9) | 4 (5.0) |
|  | Malignancy/Cancer | 11 (3.9) | 7 (3.4) | 4 (5.0) |
|  | Short life expectancy | 11 (3.9) | 10 (4.9) | 1 (1.3) |
|  | Chronic kidney disease | 10 (3.5) | 5 (2.5) | 5 (6.3) |
|  | American Society of Anaesthesiology (ASA) score | 10 (3.5) | 7 (3.4) | 3 (3.8) |
|  | Previous hip fracture | 10 (3.5) | 7 (3.4) | 3 (3.8) |
|  | Other patient barriers* | 5 (1.8) | 5 (2.5) |  |
| Process | Type of surgery (e.g., fixation, hemi-arthroplasty, arthroplasty) | 63 (22.2) | 43 (21.1) | 20 (25.0) |
|  | Surgical approach (e.g., anterior, anterolateral, posterior) | 22 (7.7) | 12 (5.9) | 10 (12.5) |
|  | Type of anaesthesia | 19 (6.7) | 12 (5.9) | 7 (8.8) |
|  | Time to surgery | 16 (5.6) | 8 (3.9) | 8 (10.0) |
|  | Other process barriers† | 3 (1.1) | 3 (1.5) | 0 |
| Structure | Staffing issues (availability of staff to support) | 55 (19.4) | 31 (15.2) | 24 (30.0) |
|  | Insufficient resources/equipment | 39 (13.7) | 22 (10.8) | 17 (21.3) |
|  | Low multidisciplinary engagement | 32 (11.3) | 16 (7.8) | 16 (20.0) |

* Included bone quality, unstable patients and outdated misconceptions

† Included conflicts between surgical instructions and protocols, specific surgical directions, and gaps in communication leading to improvised restrictions.
